# Influenza: The Latest Memorandum

**Published:** 1919-03-01

**Authors:** 


### Influenza: the Latest Memorandum.

THE precautionary measures advocated in the recent publication of the Local Government Board are sound and clear for public understanding, and should do much to dispel some of the confusion caused by an enthusiastic but not always correctly informed lay press. Most of the described procedures aim, however, to prevent an infection directly conveyed by secretions from the respiratory passages, and insufficient stress is laid upon the possibility of indirect transference by water contamination. In America recent observations have been made by competent authorities in camps containing 66,000 men, and proved that the incidence of and death rate from influenzal pneumonia were markedly influenced by the washing of all mess equipment in sterilised water. In battalions taking the precaution first to immerse everything in boiling water, the influenza rate was twelve times less than in those content with the usual luke-warm method of cleansing. This is a small aspect of the water question, but suggests a point deserving of closer attention.

On the subject of protective inoculation, the official advice justifiably states that, being uncertain of the exact primary cause of the disease, it is impossible to guarantee artificial immunity to infection, but that much should be possible in warding off the attacks of secondarily invading organisms. Although bacteriological opinion is not undivided, yet for several years it has been accepted that considerable resistance may be developed in most individuals by graduated inoculation against mixed strains of pneumococci and streptococci. It is very certain that such organisms are responsible for a high percentage of influenzal complications, and though, as stated by a contemporary, vaccine treatment may often be a shot in the dark, we feel that it is so often a successful one that the method is more than justified. The same writer objects that the initial dose may cause a dangerous reaction at a time when infection is rife and the patient needs his full bodily vigour. But surely in experienced hands the dosage would be such as to cause a normal but not a dangerous reaction, and in any case, during this negative phase, every precaution would be taken to safeguard and restrain the individual from encounter with virulent influenzal infections. The facts noted in the memorandum that persons attacked in the summer of 1918 have suffered less than others in the autumn epidemics, and that among them, when re-infected, complications were relatively rare, lead one confidently to hope for the ultimate success of a protective vaccine treatment and artificial immunity.

